# Biocontrol strategies: an eco-smart tool for integrated pest and diseases management

**DOI:** 10.1186/s12866-022-02744-2

**Published:** 2022-12-30

**Authors:** Durgesh Kumar Jaiswal, Suresh Janardhan Gawande, P. S. Soumia, Ram Krishna, Anukool Vaishnav, Avinash Bapurao Ade

**Affiliations:** 1 Department of Botany, Savitribai Phule Pune University, Pune, Maharashtra India; 2 ICAR-Directorate of Onion and Garlic Research, Rajgurunagar, Pune India; 3 ICAR- Indian Institute of Vegetable Research, Varanasi, Uttar Pradesh India; 4 University of Zurich, Reckenholzstrasse, Zurich, Switzerland

**Keywords:** Plant disease, Pest, Bio-control agents (BCA), PGPM, Entomopathogenic microorganism pathogenesis related proteins (PRs), Induced systemic response (ISR), Sustainable agriculture

## Abstract

For the burgeoning global population, sustainable agriculture practices are crucial for accomplishing the zero-hunger goal. The agriculture sector is very concerned about the rise in insecticide resistance and the Modern Environmental Health Hazards (MEHHs) that are problems for public health due to on pesticide exposure and residues. Currently, farming practices are being developed based on microbial bio-stimulants, which have fewer negative effects and are more efficient than synthetic agro-chemicals. In this context, one of the most important approaches in sustainable agriculture is the use of biocontrol microbes that can suppress phytopathogens and insects. Simultaneously, it is critical to comprehend the role of these microbes in promoting growth and disease control, and their application as biofertilizers and biopesticides, the success of which in the field is currently inconsistent. Therefore, editorial is part of a special issue titled "Biocontrol Strategies: An Eco-smart Tool for Integrated Pest and Disease Management" which focuses on biocontrol approaches that can suppress the biotic stresses, alter plant defense mechanisms, and offer new eco-smart ways for controlling plant pathogens and insect pests under sustainable agriculture.

## Backgrounds

By 2050, there will be 10 billion people on the planet, and feeding them is the biggest challenge facing global agriculture [1, 2]. Plants are the only direct source that can supply humans with 90% of their calories and 80% of their protein. To meet rising global demand, food production is currently being increased in earnest on a worldwide scale [3, 4]. Biotic and abiotic stressors are the main barrier to sustainable food production. These issues have recently grown to be of great concern on a global scale [5, 6]. The yearly economic loss from biotic stressors is $40 billion and results in crop losses of 20–40% [4, 7, 8]. Many serious social issues were reported due to the infestation of pathogens in food crops like *Phytophthora infestans* pathogen, responsible for potato late blight, which wiped out a million Irish people and forced another 1.5 million to leave their homes in the 1840s; it left an indelible mark on human history [9]. Another classical case of late blight causes an annual loss of $6.7 billion to the potato industry.

Similarly, due to the rice brown leaf spot disease caused by *Helminthosporium oryzae*. Many serious social issues were reported due to the infestation of pathogens in food crops like Phytophthora infestans, which caused potato late blight wiped out almost a million Irish people and forced another 1.5 million to flee their homes in the 1840s, and left an indelible mark on human history [9]. According to conservative estimates, the potato sector suffers a yearly loss of $6.7 billion due to the late blight, as does the rice industry from brown leaf spots caused *by H. oryzae*. Two million people were estimated to die during the 1940s due to the devastating famine in Bengal, which negatively influenced rice output. The corn leaf blight pandemic caused by *Helminthosporium maydis* devastated 15% of the maize harvest in the United States and cost an estimated $1 billion in 1970 [10, 11]. The catastrophic effects of pandemic plant pests have affected all the continents of the world. Therefore, efficient and eco-friendly disease management tools are pre-requisite for the global food, fiber, and biomaterials supply chains [12].

Microbiologists, plant pathologists, and entomologists across the globe face a significant challenge as they work to find and develop environmentally friendly control agents against plant diseases & pests. Their goal is to reduce the widespread use of chemical pesticides, which would be an important step forward. On the other hand, pesticides and biopesticides derived from beneficial microorganisms are among the most effective strategies for risk-free crop management during low to medium biotic stresses. Numerous early publications [13–18] and reviews [19–23] on this issue have been published, reflecting the continually expanding interest in this field of study [24–26]. Additionally, due to the alarming rise in recent pathogen alerts and concerns about food security, all major agribusiness corporations are now investing in developing biological applications [27–29]. Researchers have concluded that biological control will remain indispensable and play a significant role in modern agriculture. A decline in biocontrol adoption around 2000 years has given way to significantly increased adoption in the last five years, largely due to supplementary biological control [30, 31], for which the political changes in Latin America, Asia, and Europe are to blame. Due to this, from 2017 to 2021, the amount of crop protection chemicals used globally dropped from 2.75 to 2.66 million metric tones (https://www.statista.com/statistics/1263077/global-pesticide-agricultural-use/).

Increased consumer demands due to the awareness created by researchers, academicians, and non-governmental organizations have all been hastening this shift. Growing educational opportunities in plant protection training over the past few decades have led to the widespread successful use of biological control, particularly in developing countries like Brazil, where research and implementation of both augmentative and classical biocontrol are gaining momentum. This trend has been accelerated by the demands due to the awareness created by researchers, academicians, and non-governmental organizations that have all been hastening this shift. Growing education opportunities in plant protection training over the past few decades have resulted in the successful use of biological control on a large scale, especially in developing nations like India, China & Brazil, where research and implementation of both augmentative and classical biocontrol are gaining momentum. Realizing that synthetic pesticides and fertilizers have damaged ecosystems and exacerbated food security concerns, China and India have invested in biological control research, training, and adoption [31–33].

## Challenges

The growing concerns about the overuse of synthetic chemical pesticides and their residues, increased significance of insect pests and pathogens due to increased food demand, the withdrawal of several chemical pesticides, including soil fumigants, the appearance of new invasive species, and pesticide-resistant strains of pests, climate change, and specialized monoculture are all factors have contributed to the expansion of the biological control domain of plant protection under the sustainable agriculture goal. However, bio-control agents (BCAs) have advantages over traditional crop protection (CPs) methods but are not yet ready to take their place. In many cases, the adaptability of BCA in a non-native environment is poor. Further, their efficacy against multiple pathogens/insect pests is also low. As a result, it hasn't been widely used [34].

Another major challenge is the lack of adequate characterization of bio-agents coupled with the poor marketing strategy of bioagent-producing firms; for example, many PGPRs/biofertilizers projected as a biocontrol agent; low efficacy in non-native soils/environment work on adaptability, and its contains is lacking appropriate research on the efficacy, growth promoting activities; lacking response of bio-agents at the physiological and molecular level, and poor of characterization and product formulation of bio-agents. The mild disease/pest suppression by these bio-agents may probably be due to their growth-promoting effect on plants. Therefore, this product confuses end users because they expect anti-pest activity from these products. As a result, they are often ineffective, which helps diminish microbial biocontrol agents' reputation. Even though many strains blur the distinction between plant protection products and biopesticide/biofertilizers, strict regulation is required to ensure the efficacy of biopesticide microorganisms and prevent their misuse as plant protection products. Presently, only a few genera, species, and strains of BCAs (*Coniothyrium minitans*, *Gliocladium catenulatum*, *Pseudomonas chlororaphis* and spp., *Streptomyces griseovirides* and *Streptomyces lydicus*, and *Trichoderma asperellum*, *T. atroviride*, and *T. harzianum*) are registered against some soil-borne pathogens. Similarly, *Bacillus firmus* and *Purpureocillium lilacinum* are the only BCAs approved for use against nematodes [35].

Experts have encountered great difficulties in developing various BCA products, in addition to the cost and scalability challenges of BCA. Many alternative solutions, such as those based on fermentation or pheromones, are prohibitively expensive to manufacture, providing customers with a little financial incentive to switch away from using known BCA-containing products. As a result, several companies are looking into novel ways to reduce production costs. Due to the low barriers to entry and high market attractiveness in this domain, hundreds of companies, ranging from major CP firms to many mid-tier firms, engage in the BCA and bio-stimulants industries. Many new businesses emerge due to the influx of venture capital, but they frequently lack the funds to register their company, develop their products, and enter the market [34]. To register a single strain for commercial usage, firms would have to conduct extensive, statistically significant efficacy trials for each crop/disease in each zone. This limitation has resulted in a dearth of products for the biological control of insect pests and diseases in Europe and Asia. As was previously stated, only a handful of these products have been approved for use by European growers [35].

## Opportunities

Biological control is a cost-effective, eco-friendly, and long-term solution for crop protection against biotic stresses. Progressive farmers increasingly use the conservation and management of endangered species of biocontrol microorganisms, among other biologicals, to combat plant diseases [36]. The most successful approach to biological management for conservation objectives, according to Kean et al. [37], is to concentrate on the most critical aspects of natural enemy ecology. According to Heimpel and Mills [38], there are two strategies to boost natural enemy effectiveness: (1) changing the habitat so that natural enemies benefit at the expense of pests or (2) decreasing the detrimental effects of pesticides on natural enemies. Furthermore, the significance of biological control conservation in developing countries has been emphasized [31, 39]. Numerous microorganisms have been shown to be effective against soil-borne diseases and nematodes over the last 50 years. Among these are the active ingredients in at least one biopesticide that is already on the market. Even though several of these strains were developed a few years ago, none have achieved widespread commercial success due to competition from synthetic chemical fumigants, which are often more cost-effective, easier to apply, have a wider spectrum of activity, and are highly effective. Since the ban on methyl bromide and other chemicals, there has been a revived interest in microbial biocontrol agents against soil-borne diseases. These agents operate best in conjunction with other agronomic practices or resistant/tolerant plant varieties. The mechanism of action of microbial biocontrol agents against plant pathogens includes direct antibiosis, hyper-parasitism, resistance induction, and competition for space and nutrients.

In addition, researchers are investigating the role of non-pathogenic beneficial rhizobacteria in increasing plant resistance to pathogens, a process known as induced systemic resistance (ISR). Plant pathogen infection can result in systemic acquired resistance (SAR) [4, 40–43]. Some microorganisms (such as *Bacillus* spp*., Pseudomonas* spp., *Acinetobacter calcoaceticus*; *Azotobacter* spp., *Azospirillum* spp., *Mesorhizobium*, *Bradyrhizobium*, *Burkholderia*) act as bio-stimulators by producing indole-acetic acid, nitrogen fixation, P-solubilizing, siderophore, HCN production, 1-aminocyclopropane-1-carboxylate (ACC) deaminase, degrading organic matter to improving the plant growths & yields, controlling disease & pest and maintaining soil health's [44–48]. Furthermore, soil and plant microbiomes can act as inoculants, aid in nutrient absorption, biocontrol products, help protect plants from pests and diseases, or both. Some soil amendments may be required to ensure beneficial microbes' survival. Perhaps "probiotics" can be identified to maintain plant microbiomes healthy [49–53].

## Priorities for research in exploring of biocontrol agents

Basic biological research, particularly in taxonomy, ecology, and behavior, has tremendously aided procedures employed in the exploration, selection, and risk evaluation of biological control agents. However, some questions remain unanswered in the field of biocontrol, such as the lacking efficacy in profiling plant-associated microbial bio-controlling agents, the lack of an overall understanding of a pathogen's biology, and the epidemiology of the resulting disease, which hinder the development of disease and pest management strategies. Therefore, scientists and researchers must keep the following goals in mind as they investigate emerging issues in the field in order to establish an eco-friendly bio-control strategy.

- ▪ Exploration of a new generation of biocontrol agents with higher efficacy, high productivity in fermenters, long shelf life and the ability to be stored at room temperature, and high compatibility with other control methods
- ▪ Standardizing of identification of BCA protocol against soil-borne disease & pest 
- ▪ Population genetics research presents opportunities to better understand how the impact of biological control can be optimized.
- ▪ Improving microbiological control by integrating several strains of the same genus specie, or several genus-specie
- ▪ Increase our understanding of microbial biocontrol agents' potential against other soilborne pathogens beyond those listed on labels, as well as their potential use with carriers that can increase survival in soil, in order to demonstrate their environmental safety.
- ▪ Exploring cutting-edge genomic tools like CRISPR genome editing can reduce fewer desirable traits in biological control agents and insert new desirable characteristics such as insecticide resistance.
- ▪ Implementation of IPM strategies which include the use of microbial biocontrol agents with other management strategies 
- ▪ Optimize and reduce the cost of production of BCA by improving the technologies of fermentation or use of low-cost carrier substrates for BCA
- ▪ To protect food and ornamental crops from pathogens, it is important to encourage and assist businesses in registering microbial products that meet the criteria of 'low-risk substances' either by expediting the registration of low-risk substances or by providing subsidies to farmers who choose low-risk substances). 

## Conclusions

We believe that understanding the effective biocontrol agents and their combined impact on emerging pathogenesis and cytotoxicity requires a holistic approach of resilience and responsiveness. Furthermore, it is critical to learn about eco-friendly tools and identify viable crop protection management practices in organic and sustainable farming. Therefore, researchers are encouraged to submit papers or reviews addressing the aforementioned challenges, opportunities, and priorities for BCA research, and we also encourage researchers to submit research papers or reviews dealing with these areas: how biocontrol microbes regulate plant defense mechanisms?; deploy biocontrol actions in plants and offer new strategies for controlling plant pathogens and pests; how do plants interact with beneficial microbes while restricting pathogens?; engineering biocontrol microbial consortium and their efforts to improve, facilitate, and maintain long-term pest and disease management, as well as plant growth, human risk evaluation of rhizospheric and entomopathogenic microbes to be employed as plant pest control research on the topic of Biocontrol strategies: An Eco-smart tool for integrated pest & diseases management.

## References

[1] Krishna R, Karkute SG, Ansari WA, Jaiswal DK, Verma JP, Singh M (2019). Transgenic tomatoes for abiotic stress tolerance: status and way ahead. 3 Biotech.

[2] Jaiswal DK, Krishna R, Chouhan GK (2022). Bio-fortification of minerals in crops: current scenario and future prospects for sustainable agriculture and human health. Plant Growth Regul.

[3] Godfray HCJ, Beddington JR, Crute IR, Haddad L, Lawrence D, Muir JF (2010). Food security: the challenge of feeding 9 billion people. Science.

[4] Ab Rahman SFS, Singh E, Pieterse CM, Schenk PM (2018). Emerging microbial biocontrol strategies for plant pathogens. Plant Sci.

[5] Ingram J (2011). A food systems approach to researching food security and its interactions with global environmental change. Food Secur.

[6] Keinan A, Clark AG (2012). Recent explosive human population growth has resulted in an excess of rare genetic variants. Science.

[7] Savary S, Ficke A, Aubertot JN, Hollier C (2012). Crop losses due to diseases and their implications for global food production losses and food security. Food Secur.

[8] Soumia PS, Krishna R, Jaiswal DK, Verma JP, Yadav J, Singh M. Entomopathogenic microbes for sustainable crop protection: future perspectives. In Current trends in microbial biotechnology for sustainable agriculture. Springer; 2021:469–97.

[9] Oerke EC, Dehne HW, Schönbeck F, Weber A. Crop production and crop protection: estimated losses in major food and cash crops. Elsevier; 2012. 10.1016/C2009-0-00683-7.

[10] Ullstrup AJ (1972). Impacts of the southern corn leaf blight epidemics of 1970–1971. Annu Rev Phytopathol.

[11] Bruns HA (2017). Southern corn leaf blight: a story worth retelling. Agronomy.

[12] Oerke EC, Dehne HW (2004). Safeguarding production—losses in major crops and the role of crop protection. Crop Prot.

[13] Cook RJ, Baker KF (1983). The nature and practice of biological control of plant pathogens.

[14] Papavizas GC. Biological control in crop production. In Beltsville symposia in agricultural research (USA). Allanheld, Osmun; 1981;5:461. https://agris.fao.org/agris-search/search.do?recordID=US8207448.

[15] Pal KK, Gardener BM. Biological control of plant pathogens. Plant Health Instr. 2006:1–25. 10.1094/PHI-A-2006-1117-02.

[16] Ciancio A, Mukerji KG (2007). General concepts in integrated pest and disease management.

[17] Hajek A E, Eilenberg J. Natural enemies: an introduction to biological control. Cambridge University Press. 2018. 10.1017/9781107280267.

[18] Saroj A, Oriyomi OV, Nayak AK, Haider SZ. Phytochemicals of plant-derived essential oils: A novel green approach against pests. In Natural remedies for pest, disease and weed control, Academic Press. 2020;65–79. 10.1016/B978-0-12-819304-4.00006-3.

[19] Hoitink HA, Grebus ME (1994). Status of biological control of plant diseases with composts. Compost Sci Util.

[20] Brodeur J, Abram PK, Heimpel GE, Messing RH (2018). Trends in biological control: public interest, international networking and research direction. Biocontrol.

[21] Sarethy IP, Saharan A (2021). Genomics, proteomics and transcriptomics in the biological control of plant pathogens: a review. Indian Phytopathol.

[22] Lahlali R, Ezrari S, Radouane N, Kenfaoui J, Esmaeel Q, El Hamss H (2022). Biological control of plant pathogens: a global perspective. Microorganism.

[23] Collinge DB, Jensen DF, Rabiey M, Sarrocco S (2022). Biological control of plant diseases–what has been achieved and what is the direction?. Plant Pathol.

[24] Brown ME (1974). Seed and root bacterization. Annu Rev Phytopathol.

[25] Burr TJ, Schroth MN, Suslow T (1978). Increased potato yields by treatment of seed pieces with specific strains of *Pseudomonas**fluorescens* and *P*. *putida*. Phytopathology..

[26] Suslow TV (1982). Role of root-colonizing bacteria in plant growth. Phytopathogenic Prokaryot.

[27] Fisher MC, Henk D, Briggs CJ, Brownstein JS, Madoff LC (2012). Emerging fungal threats to animal, plant and ecosystem health. Nature.

[28] Haney CH, Samuel BS, Bush J, Ausubel FM (2015). Associations with rhizosphere bacteria can confer an adaptive advantage to plants. Nature Plant.

[29] Bach E, dos Santos Seger GD, de Carvalho FG, Lisboa BB, Passaglia LMP (2016). Evaluation of biological control and rhizosphere competence of plant growth promoting bacteria. Appl Soil Ecol.

[30] van Lenteren JC, Bolckmans K, Köhl J, Ravensberg WJ, Urbaneja A (2018). Biological control using invertebrates and microorganisms: plenty of new opportunities. BioControl..

[31] Barratt BIP, Moran VC, Bigler F, Van Lenteren JC (2018). The status of biological control and recommendations for improving uptake for the future. Biocontrol.

[32] Verma JP, Jaiswal DK, Sagar R (2014). Pesticide relevance and their microbial degradation: a-state-of-art. Rev Environ Sci Biotechnol.

[33] Parra JRP, Coelho A (2022). Insect rearing techniques for biological control programs, a component of sustainable agriculture in Brazil. Insect.

[34] Shoham J (2020). The rise of biological products in the crop protection and plant nutrition markets. Outlooks Pest Manag.

[35] Pertot I, Alabouvette C, Esteve EH, Franca S (2015). The use of microbial biocontrol agents against soil-borne diseases. Retrieved from European Commission EIPAGRI Focus Group. 2015. https://ec.europa.eu/eip/agriculture/sites/agrieip/files/8_eip_sbd_mp_biocontrol_final.pdf.

[36] Azcón-Aguilar C, Barea JM (1997). Arbuscular mycorrhizas and biological control of soil-borne plant pathogens–an overview of the mechanisms involved. Mycorrhiza.

[37] Kean J, Wratten S, Tylianakis J, Barlow N (2003). The population consequences of natural enemy enhancement, and implications for conservation biological control. Ecol Lett.

[38] Heimpel GE, Mills NJ. Biological control. Cambridge University Press; 2017. 10.1017/9781139029117.

[39] Wyckhuys KA, Lu Y, Morales H, Vazquez LL, Legaspi JC, Eliopoulos PA, Hernandez LM (2013). Current status and potential of conservation biological control for agriculture in the developing world. Biol Control.

[40] Zeier J (2021). Metabolic regulation of systemic acquired resistance. Curr Opin Plant Biol.

[41] Gao H, Guo M, Song J, Ma Y, Xu Z (2021). Signals in systemic acquired resistance of plants against microbial pathogens. Mol Biol Rep.

[42] Holmes EC, Chen YC, Mudgett MB, Sattely ES (2021). Arabidopsis UGT76B1 glycosylates N-hydroxy-pipecolic acid and inactivates systemic acquired resistance in tomato. Plant Cell.

[43] Aboulila AA (2022). Efficiency of plant growth regulators as inducers for improve systemic acquired resistance against stripe rust disease caused by *Puccinia striiformis* f. sp. tritici in wheat through up-regulation of PR-1 and PR-4 genes expression. Physiol Mol Plant Pathol.

[44] Banerjee MR, Yesmin L, Vessey JK, Rai M (2006). Plant-growth-promoting rhizobacteria as biofertilizers and biopesticides.

[45] Glick BR (2014). Bacteria with ACC deaminase can promote plant growth and help to feed the world. Microbiol Res.

[46] Pérez-Montaño F, Alías-Villegas C, Bellogín RA, Del Cerro P (2014). Plant growth promotion in cereal and leguminous agricultural important plants: from microorganism capacities to crop production. Microbiol Res.

[47] Deka H, Deka S, Baruah, CK. Plant growth promoting rhizobacteria for value addition: mechanism of action. In Plant-growth-promoting rhizobacteria (PGPR) and medicinal plants. Cham: Springer; 2015:305–21. 10.1007/978-3-319-13401-7_15.

[48] Kang SM, Shahzad R, Bilal S, Khan AL, Park YG, Lee KE (2019). Indole-3-acetic-acid and ACC deaminase *producing Leclercia adecarboxylata* MO1 improves Solanum lycopersicum L. growth and salinity stress tolerance by endogenous secondary metabolites regulation. BMC Microbiol..

[49] Kim YC, Anderson AJ (2018). Rhizosphere pseudomonads as probiotics improving plant health. Mol Plant Pathol.

[50] Wang J, Li R, Zhang H, Wei G, Li Z (2020). Beneficial bacteria activate nutrients and promote wheat growth under conditions of reduced fertilizer application. BMC Microbiol.

[51] Pirttilä AM, Mohammad Parast Tabas H, Baruah N, Koskimäki JJ. Biofertilizers and biocontrol agents for agriculture: how to identify and develop new potent microbial strains and traits. Microorganism. 2021;9(4):817. 10.3390/microorganisms9040817 PMC8069042 33924411

[52] Zheng Y, Han X, Zhao D, Wei K, Yuan Y, Li Y (2021). Exploring biocontrol agents from microbial keystone taxa associated to suppressive soil: a new attempt for a biocontrol strategy. Front Plant Sci.

[53] Harutyunyan N, Kushugulova A, Hovhannisyan N, Pepoyan A (2022). One health probiotics as biocontrol agents: one health tomato probiotics. Plant.

